# Magnetic Resonance Evaluation of Tissue Iron Deposition and Cardiac Function in Adult Regularly Transfused Thalassemia Intermedia Compared with Thalassemia Major Patients

**DOI:** 10.3390/jcm13164791

**Published:** 2024-08-14

**Authors:** Antonella Meloni, Laura Pistoia, Paolo Ricchi, Filomena Longo, Valerio Cecinati, Francesco Sorrentino, Zelia Borsellino, Sergio Bagnato, Vincenza Rossi, Priscilla Fina, Ada Riva, Stefania Renne, Giuseppe Peritore, Vincenzo Positano, Filippo Cademartiri

**Affiliations:** 1 Bioengineering Unit, Fondazione G. Monasterio CNR-Regione Toscana, 56124 Pisa, Italy; antonella.meloni@ftgm.it (A.M.); laura.pistoia@ftgm.it (L.P.); positano@ftgm.it (V.P.); 2 Department of Radiology, Fondazione G. Monasterio CNR-Regione Toscana, 56124 Pisa, Italy; 3 Unità Operativa Complessa Ricerca Clinica, Fondazione G. Monasterio CNR-Regione Toscana, 56124 Pisa, Italy; 4 Unità Operativa Semplice Dipartimentale Malattie Rare del Globulo Rosso, Azienda Ospedaliera di Rilievo Nazionale “A. Cardarelli”, 80131 Napoli, Italy; paolo.ricchi@aocardarelli.it; 5 Unità Operativa Day Hospital della Talassemia e delle Emoglobinopatie, Azienda Ospedaliero-Universitaria “S. Anna”, 44124 Cona (FE), Italy; filomena.longo@ospfe.it; 6 Struttura Semplice di Microcitemia, Ospedale “SS. Annunziata” ASL Taranto, 74123 Taranto, Italy; valerio.cecinati@als.taranto.it; 7 Day Hospital Talassemici, Ospedale “Sant’Eugenio”, 00143 Roma, Italy; sorrentino.francesco@aslrmc.it; 8 Unità Operativa Complessa Ematologia con Talassemia, “ARNAS” Civico Di Cristina Benfratelli, 90134 Palermo, Italy; zelia.borsellino@arnascivico.it; 9 Ematologia Microcitemia, Ospedale San Giovanni di Dio—ASP Crotone, 88900 Crotone, Italy; krthal@libero.it; 10 Unità Operativa Complessa Ematologia, Ospedale di Cosenza, 87100 Cosenza, Italy; enza.rossi@libero.it; 11 Unità Operativa Complessa Diagnostica per Immagini, Ospedale “Sandro Pertini”, 00157 Roma, Italy; priscilla.fina@gmail.com; 12 Struttura Complessa di Radiologia, Ospedale “SS. Annunziata” ASL Taranto, 74100 Taranto, Italy; ada.riva@yahoo.it; 13 Struttura Complessa di Cardioradiologia-UTIC, Presidio Ospedaliero “Giovanni Paolo II”, 88046 Lamezia Terme (CZ), Italy; stefania.renne@virgilio.it; 14 Unità Operativa Complessa di Radiologia, “ARNAS” Civico Di Cristina Benfratelli, 90127 Palermo, Italy; giuseppe.peritore@hotmail.it

**Keywords:** thalassemia intermedia, thalassemia major, regular transfusions, magnetic resonance imaging, iron overload, cardiac function

## Abstract

**Objectives**: This multicenter, retrospective, population-based, matched-cohort study compared clinical characteristics and magnetic resonance imaging (MRI) findings, including hepatic, pancreatic, and cardiac iron levels and cardiac function, between 135 adult regularly transfused thalassemia intermedia (TI) patients (44.73 ± 12.16 years, 77 females) and 135 age- and sex-matched thalassemia major (TM) patients (43.35 ± 9.83 years, 77 females), enrolled in the Extension-Myocardial Iron Overload in Thalassemia Network. **Methods:** The MRI protocol included the quantification of hepatic, pancreatic, and cardiac iron levels (R2* technique), the assessment of biventricular function parameters (cine images), and the detection of replacement myocardial fibrosis (late gadolinium enhancement technique). **Results:** Age, sex, frequency of splenectomy and chelation, and serum ferritin levels were not significantly different (*p* > 0.05) between the two groups, but TI patients started regular transfusions significantly later (*p* < 0.0001) and showed significantly lower pre-transfusion hemoglobin levels (*p* = 0.005). No difference was found in hepatic iron levels (*p* = 0.853). TI patients exhibited significantly lower pancreatic R2* values (*p* < 0.0001), also correcting for the duration of regular transfusions, and significantly lower cardiac R2* values (*p* < 0.0001). In the receiver operating characteristic analysis, pancreatic iron was the strongest discriminator between the two diseases. Left and right ventricular end-diastolic volume indexes were significantly higher in TI than in TM patients (*p* = 0.003 and *p* = 0.046, respectively), but the correction for the duration of regular transfusions removed the disease-specific differences (*p* > 0.05). Left ventricular (LV) mass index was significantly higher in TI (*p* = 0.049), while no difference (*p* > 0.05) was found in biventricular ejection fractions and replacement myocardial fibrosis. **Conclusions:** TI patients showed lower pancreatic and cardiac iron burden and more pronounced LV hypertrophy. These differences could not be explained by the different duration of the transfusional regimen.

## 1. Introduction

Beta (β) thalassemia is a hereditary blood disorder caused by mutations in the HBB gene, which encodes the beta-globin chains of hemoglobin. The disease is characterized by reduced or absent production of beta-globin chains, leading to an imbalance between alpha and beta chains [1,2]. This imbalance results in ineffective erythropoiesis and hemolysis, causing various degrees of anemia and related complications. Based on clinical severity, three forms of thalassemia are distinguished: major, intermedia, and minor [3]. Thalassemia major (TM), also known as Cooley’s anemia, is the most severe form of the disease and is rapidly fatal unless treated with lifelong and frequent (every 2–4 weeks) red blood cell transfusions [4,5]. The second pillar of TM treatment is iron chelation therapy, primarily aimed at decreasing the iron burden in the body and preventing or delaying long-term complications associated with iron deposition in tissues [6,7,8,9], occurring due to the lack of a natural process for the excretion of excess iron [10,11,12]. Thalassemia intermedia (TI) is characterized by a clinical phenotype of intermediate severity between thalassemia minor (the asymptomatic carrier state) and TM. Clinically, TI patients exhibit a later onset of symptoms, milder anemia, and generally a better prognosis compared with TM patients. The requirement for therapeutic interventions in TI is less frequent, and patients often maintain adequate hemoglobin levels without regular transfusions [13,14]. However, regular blood transfusions become necessary for some patients at certain points in life to sustain normal growth, improve quality of life, and prevent or manage complications [15,16,17,18,19]. Importantly, TI patients may have already accumulated considerable amounts of iron before the start of regular transfusions due to the increased intestinal absorption, driven by a paradoxical suppression of hepcidin [20,21,22].

The T2* Magnetic Resonance Imaging (MRI) technique represents the non-invasive reference standard for the detection and regular monitoring of organ-specific iron deposition [23,24,25]. Relaxation rates R2* (1000/T2*) are often reported because they are directly proportional to the iron concentration [26]. While the distribution, clinical correlates, and outcomes of iron overload in regularly transfused patients with TM have been extensively studied [27,28,29,30,31,32,33,34,35,36,37,38], R2* data in regularly transfused TI patients remain limited. A few comparative studies between non-transfused and regularly transfused TI patients demonstrated that the presence of regular transfusions was associated with increased pancreatic and cardiac iron levels [39,40]. However, no study has clearly demonstrated if, due to the different rates of blood transfusions, regularly transfused TI patients can be differentiated from the TM population based on hepatic, pancreatic, and cardiac iron levels.

Despite the regular transfusion regimen, thalassemia represents a chronically anemic condition. The body compensates for the reduced oxygen-carrying capacity by increasing the blood volume (increased preload) and decreasing the peripheral vascular resistance (decreased afterload) [41,42]. These hemodynamic changes collectively enhance the ventricular pumping capacity and result in cardiac chamber dilation [43,44]. Prolonged ventricular dilation can lead to structural and functional cardiac alterations. However, the cardiac chamber dilatation and consequent systolic dysfunction are difficult to interpret in thalassemia patients on chronic transfusion therapy. TI patients start transfusions later in life than TM patients and are subjected to prolonged periods of severe anemia and extended exposure to cardiovascular remodeling. On the other hand, cardiac size and function can also be influenced by pre-existing iron overload, which is more pronounced in TM. Indeed, cardiac and vascular iron overload can initially lead to a reduction in ventricular dimensions due to the stiffening of the vessels and ventricles [45,46,47] but in end-stage disease, it can result in an increase in ventricular dimensions and a decline in systolic function [27,48]. Cardiovascular magnetic resonance (CMR) is considered the current reference standard for the assessment of cardiac function and morphology [49,50]. Data investigating the cardiac morphology of TI patients by CMR are limited and contradictory. In the study by Liguori et al., occasionally transfused TI patients demonstrated significantly higher biventricular ejection fractions (EF) and significantly lower biventricular volumes with respect to TM patients with cardiac iron overload. No difference was detected in the comparison with TM patients without myocardial iron overload [51]. Conversely, another study demonstrated significantly increased biventricular volumes and reduced EF in never/sporadically transfused TI patients compared with TM patients [52]. A very recent study demonstrated less pronounced cardiac remodeling in never/sporadically transfused TI patients compared with regularly transfused TI patients [40]. According to our knowledge, there are no studies comparing regularly transfused TI and TM patients.

CMR also represents the gold standard for the non-invasive evaluation of replacement myocardial fibrosis [53,54], which represents an independent predictor of cardiovascular complications in both TM [55] and regularly transfused TI patients [56].

The aims of this multicenter, retrospective, population-based, matched-cohort study were to compare clinical characteristics, hepatic, pancreatic, and myocardial iron levels, biventricular function parameters, and replacement myocardial fibrosis between adult regularly transfused TI and TM patients and assess the interrelationship among these parameters.

## 2. Materials and Methods

### 2.1. Study Patients

The E-MIOT (Extension-Myocardial Iron Overload in Thalassemia) project is an Italian network of 66 thalassemia centers and 15 MRI sites that employ standardized, homogeneous, and validated MRI techniques [57,58]. All centers are connected by a web-based database that houses all the patient’s laboratory, instrumental, and clinical data.

We retrospectively selected all 135 adults (>18 years old) β-TI patients (44.73 ± 12.16 years, 77 females) who had started regular transfusions (>4 transfusions per year) in early childhood or adulthood. β-TI patients were matched by age and gender to 135 β-TM patients (43.35 ± 9.83 years, 77 females), regularly transfused since early childhood and receiving chelation treatment since mid-to-late 1970s. The match was performed to account for the difference in age distributions between the TI and TM groups.

The study complied with the Declaration of Helsinki and obtained approval from the ethical committees of all the MRI sites participating in the E-MIOT project. Written informed consent was obtained from all patients.

### 2.2. Magnetic Resonance Imaging

MRI acquisition was performed using a clinical 1.5T MRI scanner [GE Healthcare (Chicago, IL, USA), Siemens Healthineers (Erlangen, Germany), and Philips Healthcare (Best, The Netherlands)] equipped with phased-array coils. Breath-holding at end-expiration and ECG-gating were employed.

**Figure 1:**
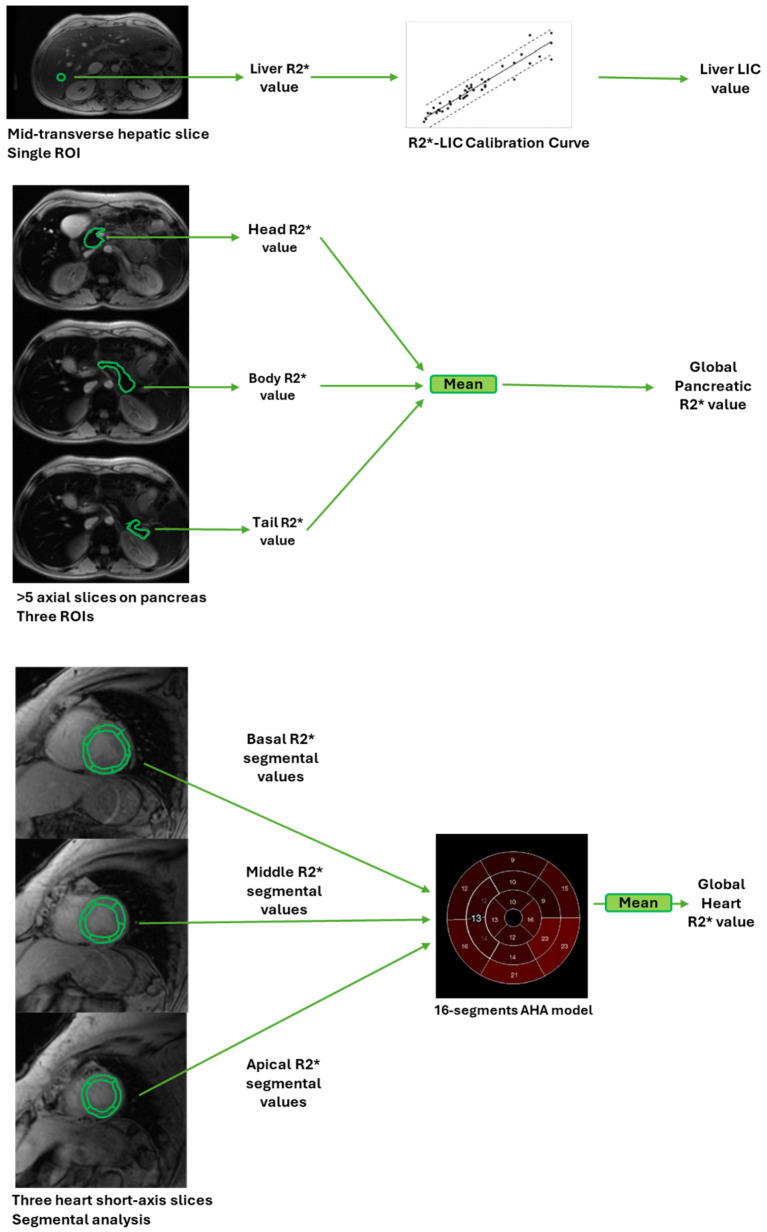
Schematic representation of hepatic, pancreatic, and cardiac iron load assessment by MRI.

T2* gradient-echo multiecho sequences were acquired for the measurement of iron overload (Figure 1). A mid-transverse hepatic slice [59], five or more axial slices including the whole pancreas [60], and three parallel short-axis views (basal, medium, and apical) of the LV [61] were acquired. A custom-written, previously validated software (HIPPO MIOT^®^, Version 2.0, Consiglio Nazionale delle Ricerche and Fondazione Toscana Gabriele Monasterio, Pisa, Italy, Year 2015) [62] was used for signal analysis on T2* images, and the derived T2* values were converted into R2* values as follows: R2* = 1000/T2*. Hepatic R2* values were determined in a circular region of interest (ROI) of standard dimension [63] and converted into liver iron concentration (LIC) values [64]. A LIC ≥ 1.8 mg/g/dw indicated hepatic iron overload [65]. Three tiny ROIs were manually delineated over the pancreatic head, body, and tail, covering the parenchymal tissue and avoiding large blood vessels, ducts, and those areas prone to susceptibility artifacts due to their close proximity to the stomach or the colonic lumen [66]. Global pancreatic R2* value was calculated as the mean of R2* values from the three regions. The highest threshold of normal global pancreas R2* value was 38 Hz [60]. The myocardial R2* distribution was mapped into a 16-segment LV standardized segmentation model, according to the American Heart Association/American College of Cardiology guidelines [67]. An appropriate correction map implemented within the software corrected for cardiac/visceral geometrical and susceptibility artifacts [62]. The global heart R2* value was obtained by averaging all segmental values. The value of 50 Hz (T2* = 20 ms) was used as a “conservative” normal value for segmental and global heart R2* values [27,62]. A global heart R2* > 50 Hz indicated significant myocardial iron overload.

To assess biventricular function parameters, cine images were acquired using a steady-state free precession sequence in two-, three-, and four-chamber planes and in the short-axis plane with whole ventricular coverage from base to apex [68]. LV and right ventricular (RV) volumes, ejection fractions, and LV mass were quantified in a standard way from short-axis cine images. Papillary muscles were included in the LV cavity volume. Biventricular volumes and LV mass were normalized for the body surface area. Cine images were also employed for the visual assessment of LV and RV regional wall motion abnormalities.

To detect replacement/focal myocardial fibrosis, late gadolinium enhancement (LGE) short-axis, vertical, horizontal, and oblique long-axis images were acquired by a T1-weighted gradient-echo inversion-recovery pulse sequence, 8–18 min after the intravenous administration of Gadobutrol (Gadovist^®^; Bayer Schering Pharma; Berlin, Germany) at the standard dose of 0.2 mmol/kg of body weight. Inversion times were adjusted to null the normal myocardium (from 210 ms to 300 ms). LGE image acquisition was not done in patients with renal impairment or who declined the administration of the contrast medium. The dichotomous presence or absence of LGE was qualitatively determined for each LV myocardial segment by reviewing all short and long axis contrast-enhanced images. Enhancement was considered present if visualized in two different spatial orientations [69].

All MRI centers joined the E-MIOT network after a validation process to ensure proper standardization of the protocol for MRI acquisition and image analysis. Good intra- and inter-operator reproducibility and inter-center transferability within the E-MIOT Network were demonstrated for the employed T2* technique [57,58] as well as for the quantification of biventricular function parameters [70].

### 2.3. Biochemical Assays

All biochemical investigations were performed using commercially available kits at the laboratories of thalassemia centers where the patients were treated.

The average value of hemoglobin and ferritin levels over the last 12 months prior to the MRI was considered.

### 2.4. Statistical Analysis

Statistical analyses were performed using SPSS version 27.0 (IBM Corp., Armonk, NY, USA) and MedCalc version 19.8 (MedCalc Software Ltd., Ostend, Belgium) statistical packages.

Continuous variables were described as mean ± standard deviation (SD), and categorical variables were expressed as frequencies and percentages.

The normality of the distribution of the parameters was assessed by using the Kolmogorov–Smirnov test or the Shapiro–Wilk test for a sample size ≤ 50.

Correlation analysis was performed using Pearson’s test or Spearman’s test as appropriate.

For continuous values with normal distribution, comparisons between two groups were made by independent-samples *t*-test. First, Levene’s test was applied to verify the homogeneity of variances (homoscedasticity). When the significance level of Levene’s test was < 0.05 and homoscedasticity could not be assumed, the Welch statistic was used. The Wilcoxon rank sum test was applied for the comparison of continuous values with non-normal distribution. Categorical variables were compared by χ^2^ test.

Analysis of covariance (ANCOVA) models were used to assess the impact of potential covariates on group differences in MRI parameters. Covariates were included if a variable was significantly different between groups and associated with the outcome being assessed. When necessary, outcomes were log-transformed to normalize the residual distributions and equalize the residual variance.

The performance of MRI parameters to discriminate regularly transfused TI patients from TM patients was assessed by receiver operating characteristic (ROC) analyses. The Youden index allowed for the depiction of optimal cut-off values from the ROC curves. Sensitivities and specificities were calculated for these cut-off values with 95% confidence intervals (CI). The Delong method was used to compare different areas under the curves (AUCs).

In all tests, two-sided *p*-values were calculated, and statistical significance was defined as *p* < 0.05.

## 3. Results

### 3.1. Demographic and Clinical Characteristics of TI and TM Patients

The demographic and clinical characteristics of the two study populations are summarized in Table 1. Age, sex, and frequency of splenectomy were comparable between the two groups, but TI patients started regular transfusions significantly later.

The frequency of chelation therapy was not significantly different between the two groups, but TI patients started chelation therapy at a significantly higher age.

The mean serum ferritin was comparable between the two groups, while pre-transfusion hemoglobin was significantly lower in TI patients.

In both the TI and TM groups, serum hemoglobin was not correlated with the duration of regular transfusions (TI: R = 0.123 *p* = 0.247; TM: R = 0.102 *p* = 0.288).

### 3.2. Clinical Correlates of Tissue Iron Levels in TI and TM

Table 2 shows the correlation of MRI LIC values, global pancreas R2* values, and global heart R2* values with demographic and clinical parameters in both TI and TM populations.

In TI, global pancreas R2* values were significantly higher in females than in males and in the splenectomized group compared with the non-splenectomized group, while hepatic and cardiac iron levels were not associated with gender or splenectomy. Only global pancreas R2* values were significantly correlated with the duration of regular transfusions. No MRI iron parameter was correlated with age or pre-transfusion hemoglobin levels, while serum ferritin levels were significantly correlated with MRI LIC values.

In TM, MRI LIC, global pancreas R2*, and global heart R2* values were all independent of gender, age, splenectomy, duration of regular transfusions, and serum hemoglobin. MRI LIC and global heart R2* values significantly correlated with serum ferritin levels.

In both the TI and TM populations, all MRI iron overload parameters were significantly correlated with each other.

### 3.3. Comparison of Tissue Iron Levels between TI and TM Patients

Table 1 shows the comparison of tissue iron levels between TI and TM patients.

No difference was found in hepatic iron levels.

The TI group was characterized by significantly lower global pancreatic R2* values and a lower frequency of pancreatic iron overload. The duration of regular transfusions was used as a covariate in the ANCOVA, and the difference in pancreatic R2* values between TI and TM remained significant (*p* = 0.020).

TI patients exhibited significantly lower global heart R2* values and number of segments with R2* > 50 Hz than TM patients. The prevalence of a significant myocardial iron overload was comparable between the two groups, but the presence of at least one myocardial segment with R2* > 50 Hz was significantly reduced in TI patients.

**Figure 2:**
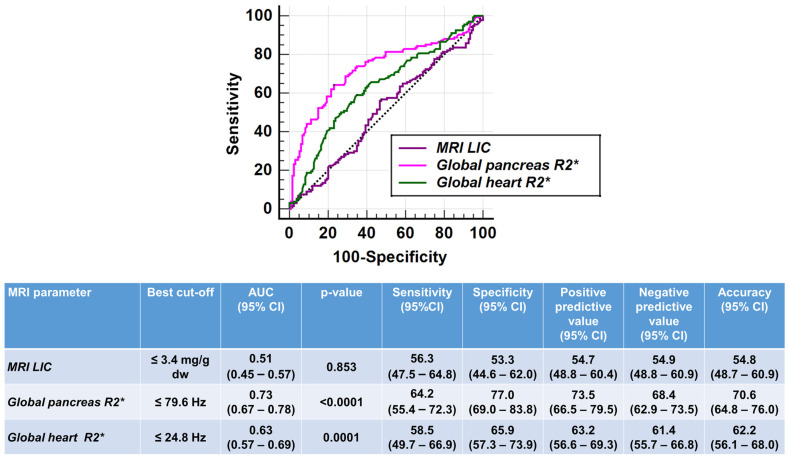
ROC curve analysis of MRI iron overload parameters to discriminate between regularly transfused TI and TM patients.

Figure 2 shows the ROC curves and the best cut-offs of MRI iron overload parameters for discriminating between regularly transfused TI and TM patients. The MRI LIC was not a discriminator and the ROC curve did not significantly deviate from the random classification (AUC = 0.51 *p* = 0.853). Both global pancreas R2* values and global heart R2* values were able to differentiate between TI and TM (AUC = 0.73 *p* < 0.00001 and AUC = 0.63 *p* = 0.0001, respectively). The Delong’s test showed a significant difference among the AUCs (*p* = 0.015), with pancreatic iron levels having a stronger discriminatory ability.

### 3.4. Clinical Correlates of Biventricular Function Parameters in TI and TM

Table 3 shows the association of biventricular function parameters with demographic and clinical parameters and iron levels in TI patients. Males and females were comparable for age (44.06 ± 14.38 vs. 45.24 ± 10.25 years, *p* = 0.596), but the male sex was associated with significantly higher biventricular end-diastolic volume indexes (EDVI) and end-systolic volume indexes (ESVI) and LV mass index and significantly lower biventricular EFs. Compared with non-splenectomized patients, splenectomized patients showed significantly higher LV EDVI and LV ESVI. Biventricular indexed volumes and LV mass index decreased significantly with age, while biventricular EFs increased significantly with age. The LV EDVI was inversely correlated with the duration of regular transfusions. No biventricular function parameter was associated with mean serum hemoglobin, mean serum ferritin, or tissue iron levels.

Table 4 shows the association of biventricular function parameters with demographic and clinical parameters and iron levels in TM patients. Males and females were comparable for age (42.14 ± 11.22 vs. 44.26 ± 8.59 years, *p* = 0.233), but the male sex was associated with significantly higher biventricular EDVI and ESVI and LV mass index and significantly lower biventricular EFs. Biventricular indexed volumes and LV mass index decreased significantly with age, while biventricular EFs increased significantly with age. With the exception of the LV mass index, all biventricular function parameters were associated with the duration of the regular transfusions. No biventricular function parameter was associated with mean serum hemoglobin, mean serum ferritin, or tissue iron levels.

### 3.5. Comparison of Biventricular Function Parameters between TI and TM Patients

Table 1 shows the comparison of biventricular function parameters between TI and TM patients.

The EDVI of both ventricles and the LV ESVI were significantly higher in TI patients than in TM patients. Serum hemoglobin or pancreatic and cardiac iron levels were not significantly associated with biventricular volumes and were not used as covariates. The ANCOVA correction for the duration of regular transfusions removed the disease-specific differences (LVEDVI: *p* = 0.626; LV ESVI: *p* = 0.288; RV EDVI: *p* = 0.951).

The LV mass index was significantly higher in TI patients than in TM patients, while the biventricular EF was comparable between the two groups.

No difference between TI and TM patients was found in the frequency of wall motion abnormalities.

### 3.6. Replacement Myocardial Fibrosis in TI and TM

The contrast medium was administered to 31.1% of TI patients and 28.9% of TM patients (*p* = 0.690). The frequency of replacement myocardial fibrosis was comparable between TI and TM patients (Table 1), and no difference was found in the number of positive-LGE segments (2.78 ± 1.48 vs. 2.82 ± 1.94, *p* = 0.937). Among the LGE areas, the septum was involved in 80.0% of the cases for TI patients and in 81.8% of the cases for TM patients (*p* = 0.916). Most patients had a non-ischemic LGE pattern, while an ischemic (transmural) pattern was found in two TM patients and one TI patient.

Table 5 shows the demographic, clinical, and MRI correlates of replacement myocardial fibrosis in both regularly transfused TI and TM patients.

In TI, only the age differed significantly between patients without and with replacement myocardial fibrosis, while no difference was found in gender, clinical characteristics, hematochemical parameters, hepatic, pancreatic, and cardiac iron levels, biventricular function parameters, or the prevalence of wall motion abnormalities.

In TM, replacement myocardial fibrosis was associated with an older age and an increased prevalence of wall motion abnormalities. No link was found with tissue iron levels or biventricular function parameters.

## 4. Discussion

We compared MRI findings between age- and sex-matched, regularly transfused adult β-TI and β-TM patients. Despite sharing a common molecular background, TI and TM present diverse disease severity and clinical phenotypes, which translate into a different diagnostic and therapeutic approach, with TI patients starting transfusions later in life and having a lower pre-transfusion hemoglobin target [71]. Indeed, at the time of the MRI examination, our TI patients exhibited significantly lower duration of transfusion treatment and pre-transfusion hemoglobin levels.

Our study showed that in transfused and chelated TI patients, both serum ferritin and hepatic iron levels were comparable to those of TM patients. It is likely that, as in TM, also in TI the transfusions can suppress the erythropoietic drive, resulting in an increase in hepcidin, the hormone that regulates the absorption and recycling of iron [72,73,74]. This may act on macrophage iron depletion, typical of non-transfused TI patients and patients with hereditary hemochromatosis, determining a preferential distribution of iron in the hepatic reticuloendothelial system and the release of ferritin into the circulation [75,76].

Besides the comparable hepatic iron levels, TI patients were characterized by lower pancreatic siderosis than TM patients and exhibited lower cardiac R2* values and number of myocardial segments with a pathological R2*. The fact that our TI and TM patients were not heavily loaded at the cardiac level prevented detection of a significant difference in terms of the prevalence of significant myocardial iron overload. However, we found out that the frequency of patients with at least one segment with R2* > 50 Hz was significantly lower in the TI group, confirming that the segmental R2* approach is highly sensitive, enabling the detection of an uneven or early cardiac iron accumulation [77] and the identification of preferential patterns of iron distribution correlated with clinical endpoints [55,61]. The disease-specific difference in pancreatic R2* values remained after ANCOVA correction for the duration of regular transfusions, which in TI patients correlated directly with pancreatic R2* values. Conversely, in both groups, cardiac R2* values were independent of the duration of regular transfusions. So, the duration of the transfusion could not be the only cause of the disparity in pancreatic and cardiac iron levels. We hypothesize that a role may be played by the differences not only in the transfusion frequency and volume but also in the degree of ineffective erythropoiesis. Since our TI patients were maintained at lower pre-transfusion hemoglobin levels, they may be characterized by higher residual erythropoietic drive and consequent hepcidin suppression [78]. Hepcidin is the main physiological regulator of transferrin saturation and non-transferrin-bound iron (NTBI), which appears in the plasma when the iron-binding capacity of transferrin is exceeded [79,80]. The kinetics of iron absorption and elimination in extrahepatic tissues diverge from those observed in the liver [81]. The pathway through which pancreatic and cardiac siderosis occur involves plasma NTBI, which is taken into these organs through L-type calcium channels [82,83,84]. Studies comparing hepcidin levels in transfused TI and TM patients are necessary to test our hypothesis.

It is important to underline that pancreatic iron levels demonstrated better discriminative ability between TI and TM compared with cardiac iron levels, likely because pancreatic iron deposition precedes cardiac siderosis [30,31,39].

As expected, due to the chronic anemia, which reduces oxygen delivery to tissues, causing a compensatory cardiovascular response [43,44], both the TI and TM groups exhibited biventricular dilation and LV hypertrophy relative to published values for healthy subjects [68,85,86,87]. In agreement with previous studies, we failed to detect an association of biventricular function parameters with pre-transfusion hemoglobin and cardiac iron levels [40,68,85,86]. The detected sex differences in biventricular function parameters are not unexpected, but they have been previously demonstrated only in TM patients [68,88], while studies specifically addressing this issue in regularly transfused TI patients are lacking.

The EDVI of both ventricles, the LV ESVI, and the LV mass index were significantly higher in TI than in TM patients. Importantly, the ANCOVA correction for the duration of regular transfusions eliminated the disease-specific differences in ventricular volumes. This finding suggests that the increased lifetime exposure to more severe anemia is a strong determinant of the more pronounced compensatory myocardial remodeling of TI patients. In TI, red blood cell transfusions are often delayed until hemoglobin levels are critically low for a long time, and the consequent prolonged state of high cardiac output puts significant stress on the cardiovascular system [71]. The clinical implication of the more pronounced cardiac remodeling of regularly transfused TI patients is the need to define “normal for regularly transfused TI” reference ranges. Reference ranges for biventricular volumes and function normalized by age and gender-specific to TM patients had already been defined [59], but their use in TI would lead to a misdiagnosis of cardiac impairment.

In regards to the replacement myocardial fibrosis, its frequency, the number of involved segments, and the prevalence of septal involvement were comparable between the two groups. In line with a previous study involving TM patients [69], replacement myocardial fibrosis emerged as an age-dependent process in both groups. We did not detect significant differences between LGE positive and negative patients in terms of cardiac iron burden and global systolic function. Different reasons may account for this result, including the small number of patients with LGE determined by the small number of patients who received the contrast medium, the presence of normal or only slightly abnormal global heart R2* values and biventricular ejection fractions in most patients, and the fact that, unlike cardiac iron accumulation and dysfunction, replacement fibrosis seems irreversible [89].

### Study Limitations

This study is not free from limitations.

We did not collect transfusion data and we could not assess the correlation of the assessed MRI parameters with the total iron intake and eventually use it as a covariate in the ANCOVA model. Variables not accounted for in the analysis may confound and bias the results and make generalizability more challenging.

Due to the different clinical indications for chronic transfusion therapy, we could not simultaneously match TI and TM patients for age, duration, or intensity of blood transfusions.

The MRI protocol did not include the assessment of myocardial T1 values and biventricular strain parameters. The T1 mapping provides greater sensitivity than T2*/R2* in detecting changes related to mild/early myocardial iron overload and it permits the detection and quantification of microscopic fibrosis [90], but it was not available in all MRI systems at the time of patient enrollment. Myocardial strain is a more sensitive marker of myocardial dysfunction than the EF [91]. Strain parameters can be quantified from routine cine images using dedicated CMR-feature tracking software [92], which is, however, not available in all the MRI centers of the E-MIOT Network.

## 5. Conclusions

Compared with TM patients, regularly transfused TI patients showed lower pancreatic and cardiac iron burden, and these differences could not be explained by the different durations of the transfusion regimen. Pancreatic iron levels emerged as a more sensitive marker to differentiate between the two patient groups when compared with cardiac iron levels. TI patients exhibited more pronounced cardiac remodeling, suggesting the need to define “normal for TI” limits of biventricular size and function. Our findings may provide a foundation to evaluate the early initiation of treatments such as red blood cell transfusions and, of consequence, chelation therapy in TI patients.

## Figures and Tables

**Table 1:** Comparisons of demographic, clinical, and instrumental data between regularly transfused TI and TM patients.

	TI Group (*n* = 135)	TM Group (*n* = 135)	*p*-Value
Age (yrs)	44.73 ± 12.16	43.35 ± 9.83	0.306
Females, *n* (%)	77 (57.0)	77 (57.0)	1.000
Splenectomy, *n* (%)	109 (80.7)	104 (77.0)	0.456
Age at splenectomy (years)	16.29 ± 10.61	13.44 ± 8.17	0.124
Age at start of regular transfusions (years)	19.19 ± 18.51	1.34 ± 1.51	<0.0001
Duration of regular transfusions (years)	25.15 ± 15.26	41.37 ± 9.62	<0.0001
Patients in chelation therapy, *n* (%)	130 (96.3)	145 (100)	0.060
Age at start of chelation therapy (years)	17.76 ± 16.19	6.41 ± 5.98	<0.0001
Mean pre-transfusion hemoglobin (g/dL)	9.49 ± 0.58	9.72 ± 0.51	0.005
Mean serum ferritin (ng/mL)	861.82 ± 947.34	762.79 ± 736.82	0.584
MRI LIC (mg/g dw)	6.54 ± 14.68	5.22 ± 6.62	0.853
Hepatic iron overload, *n* (%)	87 (64.4)	90 (66.7)	0.701
Global pancreas R2* (Hz)	93.47 ± 93.23	148.38 ± 107.73	<0.0001
Pancreatic iron overload, *n* (%)	92 (68.1)	127 (94.1)	<0.0001
Global heart R2* (Hz)	26.19 ± 7.17	30.38 ± 16.57	<0.0001
Significant myocardial iron overload, *n* (%)	4 (3.0)	7 (5.2)	0.540
n. of segments with R2* > 50 Hz	0.44 ± 2.04	1.24 ± 3.73	0.010
At least 1 segment with R2* > 50 Hz, *n* (%)	13 (9.6)	28 (20.7)	0.011
LV EDVI (mL/m^2^)	86.77 ± 16.57	81.36 ± 16.84	0.003
LV ESVI (mL/m^2^)	33.17 ± 11.80	29.49 ± 10.29	0.006
LV mass index (g/m^2^)	57.88 ± 13.28	54.63 ± 13.27	0.049
LV EF (%)	62.89 ± 7.36	64.24 ± 7.10	0.129
RV EDVI (mL/m^2^)	83.45 ± 17.49	78.96 ± 17.05	0.046
RV ESVI (mL/m^2^)	31.27 ± 12.06	29.31 ± 10.34	0.253
RV EF (%)	63.41 ± 8.15	62.61 ± 9.21	0.546
Wall motion abnormalities, *n* (%)	11 (8.1)	11 (8.1)	1.000
Replacement myocardial fibrosis, *n* (%)	10/42 (23.8)	11/39 (28.2)	0.652

TI = thalassemia intermedia, TM = thalassemia major, *n* = number, MRI = magnetic resonance imaging, LIC = liver iron concentration, LV = left ventricular, EDVI = end-diastolic volume index, ESVI = end-systolic volume index, EF = ejection fraction, RV = right ventricular.

**Table 2:** Demographic and clinical correlates of hepatic, pancreatic, and cardiac iron levels assessed by MRI in regularly transfused TI and TM patients.

	TI Patients			TM Patients		
	MRI LIC	Global Pancreas R2*	Global Heart R2*	MRI LIC	Global Pancreas R2*	Global Heart R2*
Categorical variables	Difference of MRI iron overload parameter between two groups (absent vs. present)					
Female sex	6.39 ± 19.54 vs. 6.65 ± 9.68 mg/g dw (*p* = 0.824)	69.16 ± 76.26 vs. 111.47 ± 100.76 Hz (*p* = 0.011)	25.44 ± 4.94 vs. 26.77 ± 8.46 Hz (*p* = 0.986)	5.94 ± 7.29 vs. 4.68 ± 6.06 mg/g dw (*p* = 0.363)	148.77 ± 100.35 vs. 148.08 ± 113.62 Hz (*p* = 0.922)	28.97 ± 11.29 vs. 31.44 ± 19.64 Hz (*p* = 0.769)
Splenectomy	7.36 ± 10.70 vs. 6.34 ± 15.52 mg/g dw (*p* = 0.056)	62.21 ± 70.92 vs. 100.99 ± 96.21 Hz (*p* = 0.004)	24.71 ± 2.92 vs. 26.55 ± 7.82 Hz (*p* = 0.491)	6.31 ± 7.26 vs. 4.90 ± 6.42 mg/g dw (*p* = 0.104)	121.87 ± 91.78 vs. 154.19 ± 111.78 Hz (*p* = 0.150)	28.37 ± 13.16 vs. 30.98 ± 17.47 Hz (*p* = 0.384)
Continuous variables	Correlation (R, *p*-value) with MRI iron overload parameter					
Age	R = −0.154, *p* = 0.075	R = −0.158, *p* = 0.069	R = −0.134, *p* = 0.121	R = −0.135, *p* = 0.118	R = 0.086, *p* = 0.321	R = 0.035, *p* = 0.690
Duration of regular transfusions	R = −0.197, *p* = 0.085	R = 0.253, *p* = 0.007	R = −0.061, *p* = 0.518	R= −0.144, *p* = 0.108	R = 0.148, *p* = 0.100	R = 0.057, *p* = 0.525
Pre-transfusion hemoglobin	R = −0.126, *p* = 0.200	R = 0.004, *p* = 0.972	R = 0.068, *p* = 0.491	R = −0.165, *p* = 0.075	R = −0.121, *p* = 0.095	R = −0.004, *p* = 0.970
Mean serum ferritin	R = 0.707, *p* < 0.0001	R = 0.148, *p* = 0.129	R = 0.160, *p* = 0.102	R = 0.650, *p* < 0.0001	R = 0.085, *p* = 0.367	R = 0.251, *p* = 0.007
MRI LIC		R = 0.253 *p* = 0.003	R = 0.212 *p* = 0.014		R = 0.167, *p* = 0.042	R = 0.274, *p* = 0.001
Global pancreas R2*	R = 0.253 *p* = 0.003		R = 0.306 *p* < 0.0001	R = 0.167, *p* = 0.042		R = 0.275, *p* = 0.001
Global heart R2*	R = 0.212 *p* = 0.014	R = 0.306 *p* < 0.0001		R = 0.274, *p* = 0.001	R = 0.275, *p* = 0.001	

TI = thalassemia intermedia, TM = thalassemia major, MRI = magnetic resonance imaging, LIC = liver iron concentration.

**Table 3:** Demographic, clinical, and MRI correlates of biventricular function parameters in regularly transfused TI patients.

	LV EDVI	LV ESVI	LV Mass Index	LV EF	RV EDVI	RV ESVI	RV EF
Categorical variables	Difference in biventricular parameters between two groups (absent vs. present)						
Female sex	95.52 ± 16.09 vs. 80.18 ± 13.69 mL/m^2^ (*p* < 0.0001)	38.45 ± 11.08 vs. 29.19 ± 10.79 mL/m^2^ (*p* < 0.0001)	65.91 ± 13.47 vs. 51.83 ± 9.42 g/m^2^ (*p* < 0.0001)	60.34 ± 7.78 vs. 64.82 ± 6.44 % (*p* < 0.0001)	93.97 ± 17.18 vs. 75.54 ± 13.05 mL/m^2^ (*p* < 0.0001)	38.54 ± 12.47 vs. 25.79 ± 8.31 mL/m^2^ (*p* < 0.0001)	60.10 ± 7.24 vs. 65.91 ± 7.95 % (*p* < 0.0001)
Splenectomy	79.15 ± 13.75 vs. 88.59 ± 16.72 mL/m^2^ (*p* = 0.009)	29.04 ± 9.37 vs. 34.16 ± 12.14 mL/m^2^ (*p* = 0.047)	53.42 ± 15.71 vs. 58.95 ± 12.48 g/m^2^ (*p* = 0.144)	63.46 ± 7.32 vs. 62.76 ± 7.39 % (*p* = 0.665)	78.54 ± 17.64 vs. 84.63 ± 17.33 mL/m^2^ (*p* = 0.111)	29.23 ± 12.35 vs. 31.75 ± 11.99 mL/m^2^ (*p* = 0.191	65.35 ± 8.27 vs. 62.95 ± 8.09 % (*p* = 0.337)
Continuous variables	Correlation (R, *p*-value) with biventricular function parameter						
Age	R = −0.310, *p* < 0.0001	R = −0.279, *p* = 0.001	R = −0.191, *p* = 0.027	R = 0.211, *p* = 0.014	R = −0.250, *p* = 0.003	R = −0.231, *p* = 0.007	R = 0.178, *p* = 0.039
Duration of regular transfusions	R = −0.211, *p* = 0.023	R = −0.124, *p* = 0.187	R = −0.031, *p* = 0.741	R = −0.023, *p* = 0.806	R = −0.111, *p* = 0.236	R = −0.017, *p* = 0.854	R = −0.115, *p* = 0.220
Pre-transfusion hemoglobin	R = −0.026, *p* = 0.795	R = 0.034, *p* = 0.728	R = 0.036, *p* = 0.714	R = −0.084, *p* = 0.391	R = 0.015, *p* = 0.882	R = 0.027, *p* = 0.780	R = −0.103, *p* = 0.293
Mean serum ferritin	R = 0.008, *p* = 0.936	R = 0.051, *p* = 0.601	R = 0.112, *p* = 0.253	R = −0.116, *p* = 0.236	R = −0.046, *p* = 0.636	R = −0.025, *p* = 0.801	R = 0.018, *p* = 0.857
MRI LIC	R = 0.043, *p* = 0.617	R = 0.027, *p* = 0.754	R = 0.105, *p* = 0.225	R = −0.023, *p* = 0.794	R = 0.074, *p* = 0.394	R = 0.061, *p* = 0.480	R = −0.018, *p* = 0.834
Global pancreas R2*	R = −0.081, *p* = 0.335	R = −0.116, *p* = 0.183	R = −0.079, *p* = 0.362	R = 0.121, *p* = 0.165	R = −0.040, *p* = 0.647	R = −0.072, *p* = 0.410	R = 0.065, *p* = 0.457
Global heart R2*	R = −0.006, *p* = 0.948	R = −0.042, *p* = 0.625	R = −0.036, *p* = 0.679	R = 0.090, *p* = 0.298	R = −0.052, *p* = 0.550	R = −0.098, *p* = 0.258	R = 0.165, *p* = 0.055

LV = left ventricular, EDVI = end-diastolic volume index, ESVI = end-systolic volume index, EF = ejection fraction, RV = right ventricular, MRI = magnetic resonance imaging, LIC = liver iron concentration.

**Table 4:** Demographic, clinical, and MRI correlates of biventricular function parameters in TM patients.

	LV EDVI	LV ESVI	LV Mass Index	LV EF	RV EDVI	RV ESVI	RV EF
Categorical variables	Difference in biventricular parameters between two groups (absent vs. present)						
Female sex	92.02 ± 16.66 vs. 73.34 ± 11.84 mL/m^2^ (*p* < 0.0001)	35.55 ± 10.19 vs. 24.92 ± 7.71 mL/m^2^ (*p* < 0.0001)	62.07 ± 12.92 vs. 49.03 ± 10.54 g/m^2^ (*p* < 0.0001)	61.76 ± 5.94 vs. 66.10 ± 7.37 % (*p* < 0.0001)	89.66 ± 15.09 vs. 70.91 ± 13.74 mL/m^2^ (*p* < 0.0001)	34.76 ± 10.28 vs. 25.19 ± 8.32 mL/m^2^ (*p* < 0.0001)	61.22 ± 7.46 vs. 63.66 ± 10.26 % (*p* = 0.012)
Splenectomy	80.55 ± 16.86 vs. 81.61 ± 16.91 mL/m^2^ (*p* = 0.761)	29.36 ± 9.90 vs. 29.53 ± 10.44 mL/m^2^ (*p* = 0.981)	50.77 ± 13.19 vs. 55.78 ± 13.14 g/m^2^ (*p* = 0.102)	63.84 ± 7.12 vs. 64.36 ± 7.13 % (*p* = 0.723)	77.00 ± 17.52 vs. 79.55 ± 16.95 mL/m^2^ (*p* = 0.467)	29.94 ± 11.55 vs. 29.13 ± 10.01 mL/m^2^ (*p* = 0.908)	62.19 ± 8.60 vs. 62.74 ± 9.42 % (*p* = 0.773)
Continuous variables	Correlation (R, *p*-value) with biventricular function parameter						
Age	R = −0.289, *p* = 0.001	R = −0.298, *p* < 0.0001	R = −0.044, *p* = 0.614	R = 0.223, *p* = 0.009	R = −0.363, *p* < 0.0001	R = −0.336, *p* < 0.0001	R = 0.177, *p* = 0.040
Duration of regular transfusions	R = −0.320, *p* < 0.0001	R = −0.342, *p* < 0.0001	R = −0.048, *p* = 0.597	R = 0.272, *p* = 0.002	R = −0.384, *p* < 0.0001	R = −0.357, *p* < 0.0001	R = 0.185, *p* = 0.039
Pre-transfusion hemoglobin	R = −0.181, *p* = 0.051	R = −0.107, *p* = 0.250	R = 0.035, *p* = 0.706	R = 0.003, *p* = 0.977	R = −0.105, *p* = 0.261	R = −0.072, *p* = 0.439	R = 0.030, *p* = 0.748
Mean serum ferritin	R = 0.010, *p* = 0.917	R = 0.105, *p* = 0.263	R = 0.076, *p* = 0.415	R = −0.202, *p* = 0.080	R = −0.070, *p* = 0.455	R = 0.091, *p* = 0.331	R = −0.067, *p* = 0.476
MRI LIC	R = 0.134, *p* = 0.121	R = 0.173, *p* = 0.055	R = 0.134, *p* = 0.120	R = −0.200, *p* = 0.060	R = 0.084, *p* = 0.333	R = 0.109, *p* = 0.207	R = −0.064, *p* = 0.458
Global pancreas R2*	R = −0.067, *p* = 0.438	R = −0.044, *p* = 0.615	R = 0.032, *p* = 0.709	R = 0.054, *p* = 0.536	R = −0.064, *p* = 0.464	R = −0.089, *p* = 0.306	R = −0.017, *p* = 0.844
Global heart R2*	R = −0.068, *p* = 0.432	R = −0.090, *p* = 0.298	R = −0.081, *p* = 0.352	R = 0.101, *p* = 0.245	R = −0.066, *p* = 0.445	R = −0.164, *p* = 0.057	R = 0.148, *p* = 0.086

LV = left ventricular, EDVI = end-diastolic volume index, ESVI = end-systolic volume index, EF = ejection fraction, RV = right ventricular, MRI = magnetic resonance imaging, LIC = liver iron concentration.

**Table 5:** Comparison of demographic, clinical, and MRI findings between patients without and with replacement myocardial fibrosis by LGE technique in the regularly transfused TI group and TM group.

	TI Patients			TM Patients		
	No LGE (*n* = 32)	LGE (*n* = 10)	*p*-Value	No LGE (*n* = 28)	LGE (*n* = 11)	*p*-Value
Age (yrs)	43.71 ± 11.11	50.63 ± 7.54	0.035	41.62 ± 8.31	51.41 ± 8.27	0.002
Females, *n* (%)	17 (53.1)	8 (80.0)	0.162	15 (53.6)	7 (63.6)	0.725
Splenectomy, *n* (%)	25 (78.1)	9 (90.0)	0.655	7 (75.0)	11 (100.0)	0.159
Duration of regular transfusions (years)	26.29 ± 15.36	21.09 ± 12.24	0.372	39.88 ± 8.05	49.24 ± 8.26	0.003
Patients in chelation therapy, *n* (%)	31 (96.9)	10 (100.0)	0.572	28 (100.0)	11 (100.0)	-
Mean pre-transfusion hemoglobin (g/dL)	9.46 ± 0.57	9.56 ± 0.43	0.810	9.82 ± 0.54	9.82 ± 0.49	0.773
Mean serum ferritin (ng/mL)	971.33 ± 1130.75	625.25 ± 553.49	0.408	1134.68 ± 1028.42	837.45 ± 947.53	0.117
MRI LIC (mg/g dw)	6.29 ± 9.75	5.98 ± 8.73	0.555	5.38 ± 7.11	4.60 ± 3.58	0.685
Global pancreas R2* (Hz)	75.55 ± 80.61	133.15 ± 105.99	0.140	165.22 ± 146.02	199.63 ± 136.23	0.224
Global heart R2* (Hz)	25.70 ± 6.15	24.38 ± 1.83	0.779	31.58 ± 17.60	27.25 ± 6.27	0.827
LV EDVI (mL/m^2^)	85.28 ± 18.05	80.80 ± 11.24	0.465	83.00 ± 16.54	84.36 ± 20.78	0.950
LV ESVI (mL/m^2^)	34.06 ± 12.41	30.30 ± 10.32	0.425	29.25 ± 9.00	34.55 ± 13.41	0.217
LV mass index (g/m^2^)	61.19 ± 14.95	58.50 ± 6.65	0.658	58.00 ± 14.75	59.09 ± 11.21	0.876
LV EF (%)	62.22 ± 8.26	62.60 ± 8.09	0.899	64.71 ± 6.02	58.82 ± 9.09	0.055
RV EDVI (mL/m^2^)	84.41 ± 11.17	75.80 ± 11.08	0.136	82.64 ± 15.57	81.36 ± 24.73	0.743
RV ESVI (mL/m^2^)	32.59 ± 10.39	27.90 ± 11.59	0.147	31.29 ± 9.97	35.09 ± 14.40	0.673
RV EF (%)	61.91 ± 7.05	63.40 ± 9.79	0.598	62.14 ± 6.61	56.64 ± 8.20	0.057
Wall motion abnormalities, *n* (%)	1 (3.1)	1 (10.0)	0.424	0 (0.0)	3 (27.3)	0.018

TI = thalassemia intermedia, TM = thalassemia major, LGE = late gadolinium enhancement, *n* = number, MRI = magnetic resonance imaging, LIC = liver iron concentration, LV = left ventricular, EDVI = end-diastolic volume index, ESVI = end-systolic volume index, EF = ejection fraction, RV = right ventricular.

## Data Availability

The data presented in this study are available on request from the corresponding author. The data are not publicly available due to privacy.
